# Bacterial composition and succession during storage of North-Atlantic cod *(Gadus morhua) *at superchilled temperatures

**DOI:** 10.1186/1471-2180-9-250

**Published:** 2009-12-04

**Authors:** Eyjólfur Reynisson, Hélène L Lauzon, Hannes Magnússon, Rósa Jónsdóttir, Guðrún Ólafsdóttir, Viggó Marteinsson, Guðmundur Óli Hreggviðsson

**Affiliations:** 1 Food Safety & Environment, Matis-Icelandic Food Research (Vínlandsleið 12), Reykjavík (113), Iceland; 2 Faculty of Life and Environmental Sciences, University of Iceland (Sturlugata 7), Reykjavík (107), Iceland; 3 Value Chain & Processing, Matis-Icelandic Food Research (Vínlandsleið 12), Reykjavík (113), Iceland; 4 Biotechnology & Biomolecules, Matis-Icelandic Food Research (Vínlandsleið 12), Reykjavík (113), Iceland; 5 The Icelandic Fisheries Laboratories (Skúlagata 4), Reykjavík (101), Iceland

## Abstract

**Background:**

The bacteriology during storage of the North-Atlantic cod has been investigated for the past decades using conventional cultivation strategies which have generated large amount of information. This paper presents a study where both conventional cultivation and cultivation independent approaches were used to investigate the bacterial succession during storage of cod loins at chilled and superchilled temperatures.

**Results:**

Unbrined (0.4% NaCl) and brined (2.5% NaCl) cod loins were stored at chilled (0°C) and superchilled (-2 and -3.6°C) temperatures in air or modified atmosphere (MA, % CO_2_/O_2_/N_2_: 49.0 ± 0.6/7.4 ± 0.2/43.7 ± 0.4). Discrepancy was observed between cultivation enumeration and culture independent methods where the former showed a general dominance of *Pseudomonas *spp. (up to 59%) while the latter showed a dominance of *Photobacterium phosphoreum *(up to 100%).

Gas chromatography-mass spectrophotometry (GC-MC) showed that trimethylamine was the most abundant volatile in mid- and late storage periods. Terminal restriction polymorphism (t-RFLP) analysis showed that the relative abundance of *P. phosphoreum *increased with storage time.

**Conclusion:**

The present study shows the bacteriological developments on lightly salted or non-salted cod loins during storage at superchilled temperatures. It furthermore confirms the importance of *P. phosphoreum *as a spoilage organism during storage of cod loins at low temperatures using molecular techniques. The methods used compensate each other, giving more detailed data on bacterial population developments during spoilage.

## Background

The Atlantic cod (*Gadus morhua*) is a cold-adapted fish species which has been captured for human consumption for many years. It is a perishable commodity and for that reason, preservation methods like freezing or salting have traditionally been used to extend its shelf life [1,2].

The fish is a microbial ecosystem of its own where the ecological principles of succession are as valid as in any other ecosystem. This microbiological environment consists of a high nutrient content with an oxygen tension favourable to the proliferation of fast-growing heterotrophs also responsible for the spoilage of food [3-5]. Until now, the process of fish spoilage has been investigated intensively with regard to sensory evaluation, chemical changes of volatile and non-volatile compounds and microbiological growth by cultivation methods [5-9].

*Pseudomonas *spp. and *Shewanella putrefaciens *were early recognised as putative spoilage inducers in fish muscle and have since then been found in various fish species from fresh- and marine waters as well as in other foods [10,11]. These species are generally associated with spoilage of fish stored under aerobic conditions while *Photobacterium phosphoreum *has been reported as the main spoilage organism in modified atmosphere (MA) packed fish, being CO_2_-tolerant and producing trimethylamine (TMA) from trimethylamine oxide [5,12,13]. *P. phosphoreum *is not as easily cultivated as many other heterotrophs found in fish, as it is vulnerable to temperature fluctuations [14]. The importance of this species during the spoilage of fish was therefore identified later both in MAP [12,14,15] and air-stored fish products [1,16,17]. However, storage under superchilled conditions delayed *P. phosphoreum *development in cod fillets while H_2_S-producing bacteria, most likely *Sh. putrefaciens*, were not affected and reached high levels [1]. The spoilage organisms involved in any given fish can vary among fish species and its habitat. Other bacterial species have also been associated with fish spoilage, e.g. *Brochothrix thermosphacta, Aeromonas *spp., *Vibrio *spp. and Enterobacteriaceae [8].

Until recently, most studies dealing with food microbiology of fish have used conventional cultivation methods for estimation of bacterial growth. In recent years, the use of molecular methodology has increased enormously where microbiological diversity has been documented with cultivation independent methods [18-20]. The abundance of selected species has furthermore been monitored with the use of specific detection methods such as real-time PCR [21].

The work presented here was performed in parallel to a larger shelf life trial assessing the effects of brining, MA packaging and superchilling on the shelf life and quality parameters of cod loins using conventional sensory, chemical and microbiological methods [15]. The aim of the present study was to examine the bacterial succession that occurs during storage of cod loins differently treated and stored under various conditions specifically using cultivation independent approach and compare it against conventional cultivation methods.

## Results

### Temperature and gas measurements

During the storage trials, the average ambient temperature in the three coolers was 0.0 ± 0.3°C; -2.0 ± 0.4°C and -3.6 ± 0.8°C. These groups were therefore called 0, -2 and -4°C groups. Average loin temperature in the polystyrene boxes was 0.0 ± 0.4°C (0°C air-group), -1.5 ± 1.1°C (-2°C air-group) and -2.8 ± 1.5°C (-4°C air-group). In these boxes, fish temperature of the 0°C group reached target temperature on the packaging day, the -2°C group on day 5 and the -4°C group on day 7. The average gas composition in MA packages at the beginning of the experiment was CO_2_/O_2_/N_2_: 49.0 ± 0.6/7.4 ± 0.2/43.7 ± 0.4. After 1 week storage a decrease of CO_2 _(23-28%) was detected in all packages but after that the gas composition remained essentially the same.

### Bacterial counts by cultivation during storage

**Figure 1:**
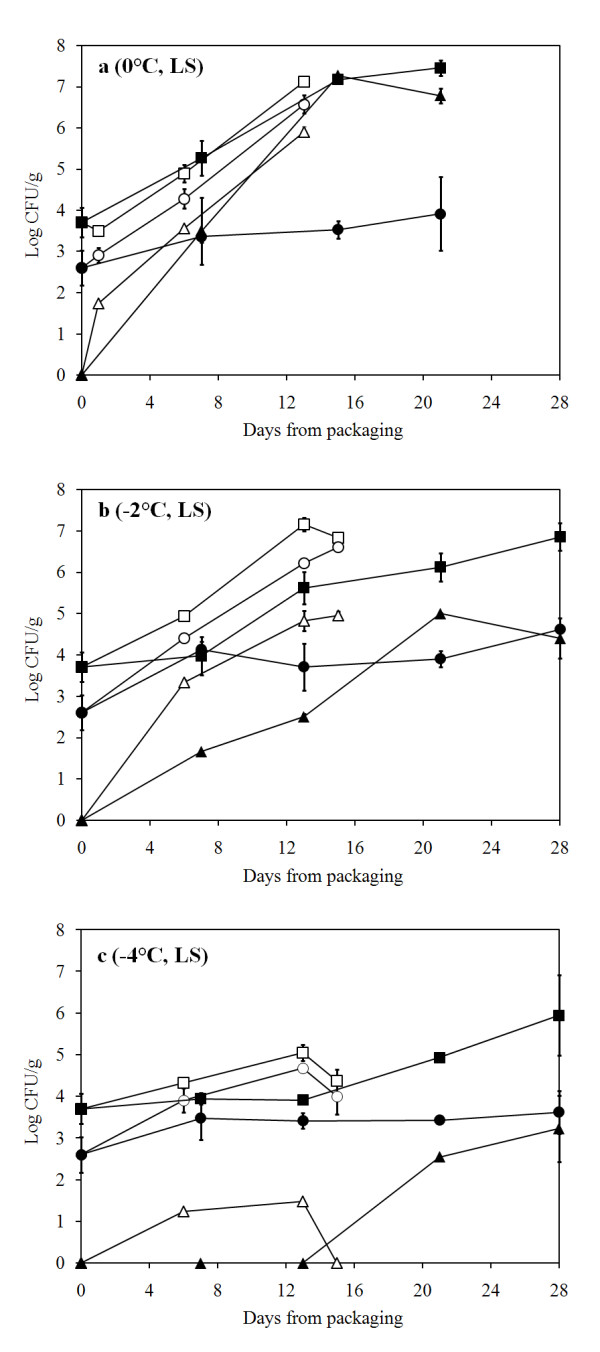
**Bacterial growth in air and MA cod loins (LS)**. Bacterial growth in air- and MA-packaged cod loins (LS) during storage at A) 0°C, B) -2°C and C) -4°C. (black square) Total psychrotrophic viable counts in MA, (white square) total psychrotrophic viable counts in air, (black circle) presumptive *Pseudomonas *counts in MA, (white circle) presumptive *Pseudomonas *counts in air, (black triangle) *P. phosphoreum *in MA and (white triangle) *P. phosphoreum *in air.

**Figure 2:**
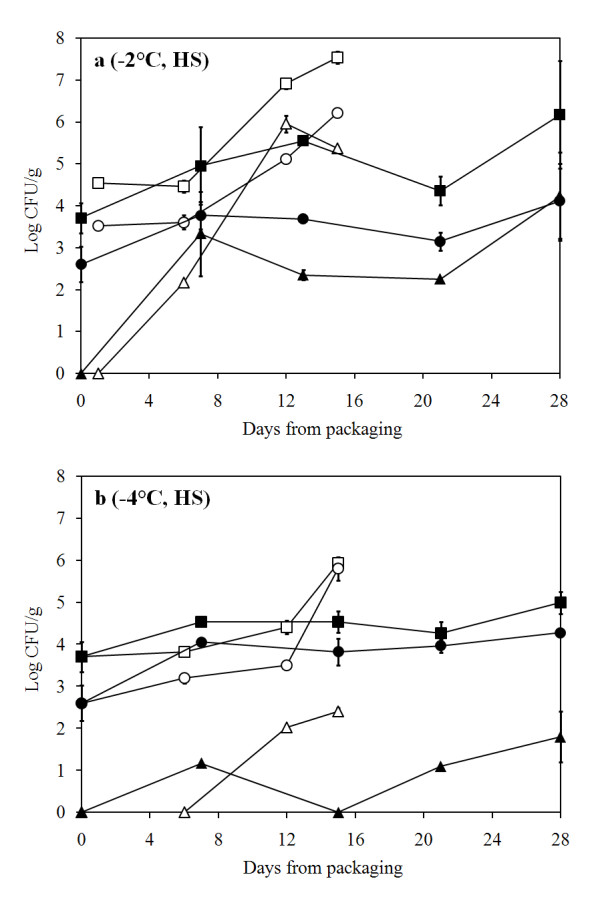
**Bacterial growth in air and MA cod loins (HS)**. Bacterial growth in air- and MA-packaged cod loins (HS) during storage at A) -2°C and B) -4°C. (black square) Total psychrotrophic viable counts in MA, (white square) total psychrotrophic viable counts in air, (black circle) presumptive *Pseudomonas *counts in MA, (white circle) presumptive *Pseudomonas *counts in air, (black triangle) *P. phosphoreum *in MA and (white triangle) *P. phosphoreum *in air.

Quality of the processed raw material (LS, low salt with 0.4% NaCl) was evaluated upon packaging and the total psychrotrophic load (TVC) was found to contain less than 10^4 ^colony forming units (CFU)/g. Initial *Pseudomonas *spp. load was tenfold lower (Fig. 1) and H_2_S-producing bacteria almost 100-fold lower than TVC (data not shown). *P. phosphoreum *was not detected (< 20 CFU/g) in newly packaged cod loins. Generally, air storage at -2°C did not inhibit bacterial growth compared to storage at 0°C whereas storage at -4°C clearly showed a reduced growth throughout the storage time (Fig. 1 and 2). In MAP fish, storage temperature clearly influenced bacterial growth, with an increased delay as temperature decreased. Monitoring of *P. phosphoreum *showed a reduction in growth with lower temperatures, especially when combined with MA (Fig. 1).

*Pseudomonas *spp. showed an increasing growth during storage in air, both at 0 and -2°C, but with some delay at -4°C. MAP had a biostatic effect on pseudomonads development, resulting in constant counts (between 3 and 4 log_10 _CFU/g) at all temperatures. Similar trends could be seen during storage of brined (HS, high salt with 2.5% NaCl) fish where combining MA and lower temperature storage generally inhibited bacterial growth (Fig. 2). Relative ratio of selected spoilage organisms showed a large variation of dominance. *Pseudomonas *spp. were usually in high proportional concentrations during air storage (up to 58.9%) and at lower concentrations during MA storage. However, on day 7 at -4°C in MA storage, *Pseudomonas *spp. reached a level of 33% of the flora in both the LS and HS groups. *P. phosphoreum *was at low relative concentrations (0 - 6%) except during MA storage at 0°C where it reached up to nearly 100% (Table 1).

**Table 1:** Relative abundance (%) of selected bacterial groups in samples from a shelf life experiment of cod loins by cultivation, 16S rRNA clone analysis and t-RFLP.

Storage conditions			Cultivation		16S	**t-RFLP**^**1**^
**Atmosphere (salt group)**	**Temp. (°C)**	**Time (days)**	***P.s***.^**2**^	***P.p***^**3**^	***P.p***^**3**^	***P.p***^**3**^

Air (LS)	-	0	7.9	Nd	0.0	0.0
Air (LS)	0	6	24.5	4.7	91.3	100
Air (LS)	0	13	28.5	6.2	95.2	84.1
Air (LS)	-2	6	29.9	2.5	61.4	40.7
Air (LS)	-2	15	58.9	1.3	93.3	86.2
Air (LS)	-4	15	42.7	Nd	83.3	100
Air (HS)	-2	6	14.0	0.5	60.0	72.3
Air (HS)	-2	15	4.8	0.7	87.8	77.1
Air (HS)	-4	15	73.3	0.03	86.0	73.1
MAP (LS)	0	7	1.2	1.7	97.4	85.5
MAP (LS)	0	15	0.02	> 99	FP	FP
MAP (LS)	0	21	0.03	21.3	100	95.1
MAP (LS)	-2	7	> 99	0.5	100	FP
MAP (LS)	-2	28	0.6	0.4	100	91.9
MAP (LS)	-4	7	34.3	Nd	100	Nd
MAP (LS)	-4	21	3.2	0.4	100	90.0
MAP (HS)	-2	13	1.4	0.1	100	94.2
MAP (HS)	-2	21	6.2	0.8	95.2	62.7
MAP (HS)	-4	7	33.5	0.04	52.5	Nd
MAP (HS)	-4	28	19.3	0.1	91.3	64.7

^1^Abundancy was calculated by dividing the peak area of the *P. phosphoreum *peak by the total peak area in the t-RFLP profile. The data was generated from analysis of reverse labelled Tf and digestion with *Alu*I.

^2^*Pseudomonas *spp.

^3 ^*Photobacterium phosphoreum*

Nd, not detected

FP, No PCR product

### Bacterial community development during storage by 16S rRNA clone analysis

Partial sequence analysis of 821 16S rRNA clones from 19 samples in the shelf life experiment was performed (Table 2). PCR and cloning of two samples failed (6 days storage in air at -4°C, for both LS and HS treatments). The identity of the closest relatives in the NCBI database had in most cases a similarity of 95-100%. In the study, 25 different bacterial species were found, with 11 of them identified to the species level, 12 to the genus level and two unclassified genera. The estimated coverage of bacteria within a sample ranged from 0.88 to 1.00 (Table 2).

**Table 2:** Relative abundance (%) of bacterial species within samples collected in the shelf life trials using 16S rRNA clone analysis.

Bacterial species/group (accession)			Air									MAP									
	
			d0	d6			d13	d15				d7				d13	d21			d28	
	
			-	0°C	-2°C	-2°C	0°C	-2°C	-4°C	-2°C	-4°C	0°C	-2°C	-4°C	-4°C	-2°C	0°C	-4°C	-2°C	-2°C	-4°C
	
			-	LS	LS	HS	LS	LS	LS	HS	HS	LS	LS	LS	HS	HS	LS	LS	HS	LS	HS
*Photobacterium phosphoreum *(DQ099331)			-	91	61	60	95	93	83	88	7	97	100	100	53	100	100	100	95	100	91
*Photobacterium indicum *(AY771742)			-	-	-	-	-	-	-	-	79	-	-	-	-	-	-	-	-	-	-
*Photobacterium profundum *(CR378665)			-	-	-	-	-	-	2	-	-	-	-	-	-	-	-	-	-	-	-
*Sphingomonas *spp. (EF462462)			42	-	-	-	-	-	-	-	-	-	-	-	-	-	-	-	-	-	-
*Bradyrhizobium *spp. (AB291825)			9	-	-	-	-	-	-	-	-	-	-	-	-	-	-	-	-	-	-
*Pseudomonas *spp. (various accession)^1^			2	2	-	-	5	-	10	-	-	-	-	-	3	-	-	-	-	-	-
*Pseudomonas fluorescens *(EF424136)			36	2	-	-	-	-	-	-	-	-	-	-	-	-	-	-	-	-	-
*Pseudomonas tolaasii *(EF111117)			-	-	5	-	-	-	-	-	-	-	-	-	-	-	-	-	-	-	-
*Acinetobacter *spp. (AF500327)			-	2	2	-	-	2	-	-	-	-	-	-	5	-	-	-	-	-	-
*Variovorax *sp. (EF471221)			11	-		-	-	-	-	-	-	-	-	-	-	-	-	-	-	-	-
*Janthinobacterium lividum *(EF204211)			-	-	11	-	-	-	-	-	-	-	-	-	-	-	-	-	-	-	2
*Psychrobacter *spp. (AM491457)			-	-	20	2	-	-	-	-	2	-	-	-	-	-	-	-	-	-	-
*Psychrobacter arcticus *(CP000082)			-	-	-	-	-	-	2	-	-	-	-	-	-	-	-	-	-	-	-
*Vibrio logei *(AY771721)			-	-	-	18	-	-	2	12	-	-	-	-	-	-	-	-	2	-	-
*Moritella *spp. (various accession)^2^			-	-	-	2	-	-	-	-	5	-	-	-	-	-	-	-	-	-	-
*Moritella marina *(AB038033)			-	-	-	11	-	-	-	-	-	-	-	-	-	-	-	-	-	-	-
*Shewanella *spp. (AB183502)			-	-	-	4	-	2	-	-	2	-	-	-	3	-	-	-	2	-	-
*Shewanella benthica *(AB008796)			-	-	-	-	-	-	-	-	-	-	-	-	-	-	-	-	-	-	4
*Pseudoalteromonas *spp. (EF156750)			-	-	-	2	-	2	-	-	5	-	-	-	-	-	-	-	-	-	-
Uncultured bacterium (EF378155)			-	2	-	-	-	-	-	-	-	3	-	-	-	-	-	-	-	-	-
*Chryseobacterium *spp. (AY536547)			-	-	-	-	-	-	-	-	-	-	-	-	20	-	-	-	-	-	-
*Flavobacterium *sp. (various accession)^3^			-	-	-	-	-	-	-	-	-	-	-	-	10	-	-	-	-	-	2
*Acidovorax *spp. (AM286541)			-	-	-	-	-	-	-	-	-	-	-	-	3	-	-	-	-	-	-
Uncultured alpha proteobacterium (AB074649)			-	-	-	-	-	-	-	-	-	-	-	-	3	-	-	-	-	-	-
*Massilia aurea *(AM231588)			-	-	-	-	-	-	-	-	-	-	-	-	3	-	-	-	-	-	-

**Total sequences analysed**			**45**	**46**	**44**	**45**	**42**	**45**	**42**	**41**	**42**	**39**	**42**	**47**	**40**	**37**	**46**	**42**	**42**	**48**	**46**
Coverage (C)			98	91	98	93	100	93	93	100	95	97	100	100	88	100	100	100	95	100	96

^1 ^Accession numbers of *Pseudomonas spp*. sequences: EF111250, AF451270, EF451774, DQ777728, EF076789, EF061900

^2 ^Accession numbers of *Moritella spp*. sequences: EF192283, DQ492814, AB120661

^3 ^Accession numbers of *Flavobacterium spp*. sequences: DQ857026, DQ640006, AM689970

In general, the analysis revealed a high dominance of *Photobacterium *in all samples except in newly packaged cod loins (LS) where it was not detected. At packaging, the microflora of cod loins was dominated by *Sphingomonas *spp. and *Ps. fluorescens *while *Variovorax *spp. and *Bradyrhizobium *spp. were present at lower levels (Table 2).

A trend towards the succession of *P. phosphoreum *with time during storage was seen in all storage conditions. Slower succession of *P. phosphoreum *was observed in samples stored in air than in MA. After six days of aerobic storage, the dominance of *P. phosphoreum *was between 60 and 71% and other bacterial species were present in lower numbers, e.g. *Pseudomonas *spp., *Shewanella *spp., *Acinetobacter *spp., *Psychrobacter *spp., *Vibrio logei*, *Moritella *spp., and *Pseudoalteromonas *spp. After further storage (13-15 days), near the end of shelf life, *P. phosphoreum *increased its relative dominance up to 83-95% of the population (Table 2).

The bacterial flora of fish stored under MA was dominated by *P. phosphoreum*, reaching levels of 91-100% of the population at all sampling times with one exception (day 7, MAP, -4°C, HS cod loins) where the dominance was 53% with other species in high relative quantity, including *Chryseobacterium *and *Flavobacterium *spp. (20 and 10%, respectively). When the same group had been stored for 28 days the bacterial flora was composed of 91% *P. phosphoreum *(Table 2).

**Figure 3:**
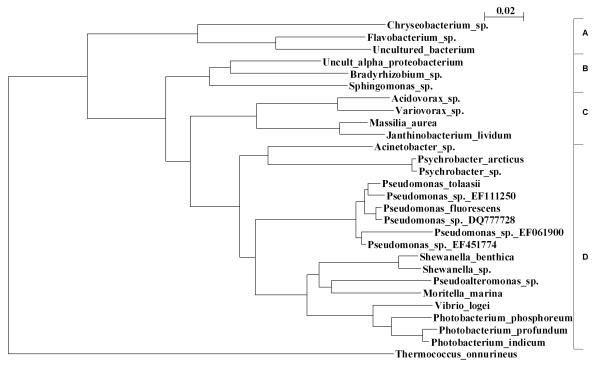
**Phylogenetic dendrogram of bacterial species**. Evolutionary neighbour-joining phylogenetic dendrogram of the 16S rRNA partial sequencing data derived from bacteria found in the shelf life experiment of cod loins. The sequence data generated from the samples was analysed and blasted on the NCBI server. The closest relative was used for construction of the dendrogram. The tree is composed of 668 bp fragments of the 16S rRNA gene and from 821 underlying sequences which were clustered in one group if the similarity was greater or equal to 98%. *Thermococcus onnurineus *was used as an outgroup. The vertical bar on the right separates the different phylogenetic classes. A: Bacteroidetes, B: Alphaproteobacteria, C: Betaproteobacteria, D: Gammaproteobacteria.

Most of the bacteria detected belong to the Gammaproteobacteria division, including the SSOs, while Bacteriodetes, Alphaproteobacteria and Betaproteobacteria had also representatives in the bacterial flora. Phylogenetic relationship between the species detected in the 16S rRNA analysis from all samples was depicted along with the division classification (Fig. 3).

### Bacterial diversity during storage by t-RFLP

**Figure 4:**
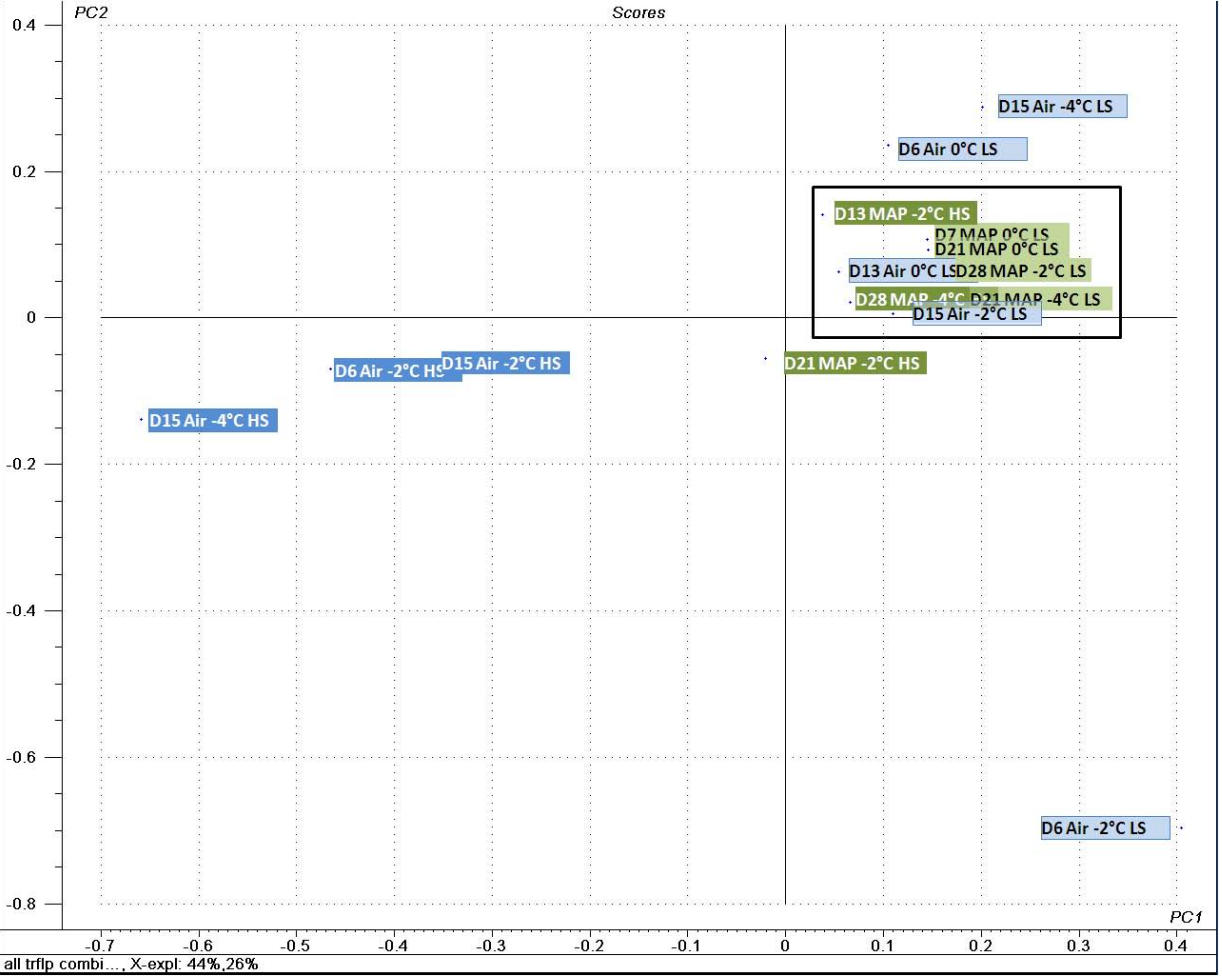
**Principal component analysis of t-RFLP datasets**. Principal component analysis of t-RFLP datasets of the bacterial flora derived from periodic sampling of cod loins in a shelf life experiment. Labels correspond to days of storage (e.g. D6 for six days), packaging method (Air or MAP), storage temperature (0, -2 or -4°C) and salt content (low salt, LS and high salt, HS). A box has been added around the samples clustering tightly together for clarification.

The number of operating taxonomic units (OTU) was different between combinations of labelled primers and restriction enzymes. Analysis of forward Tfs coupled with *Hae*III digestion resulted in 12 OTUs compared to 13 OTUs with *Alu*I in all samples analysed. The reverse Tfs coupled with *Hae*III or *Alu*I digestion resulted in 12 and 17 OTUs, respectively. Principal component analysis (PCA) of Tf profiles showed a clear difference of the bacterial flora of newly packaged cod loins (LS, day 0) compared to other samples (data not shown). Excluding the d0-samples from the analysis, a better resolution between other samples in the study was established (Fig. 4). It showed that all samples stored under MA clustered tightly together regardless of storage time, temperature or salt content. The samples stored under air showed a trend towards the MAP cluster with the exception of the 3 HS samples being situated at an opposite position in the score plot. Therefore, the first principal component (PC1) distinguishes the air-stored HS samples from other samples while PC2 seems to separate the air-stored LS samples from early storage time (D6 Air -2° LS) to late storage (D15 Air -2° LS), where the latter clusters with other air-stored and MAP samples (Fig. 4).

In this analysis PC1 explained 44% of the variability and PC2 26%. Except for the d0-samples, *P. phosphoreum *dominated the bacterial flora in all samples, with 40.7% up to 100% abundance (Table 1) when analysing the t-RFLP profile of labelled forward primer and *Alu*I digestion. Using this primer/restriction enzyme combination and analysing the sequence data of the clone library it was evident that a peak of 336 base pairs derived only from *P. phosphoreum *and *Vibrio logei*. Other combinations had more species with common terminal restriction site as *P. phosphoreum*.

### Gas Chromatography-Mass spectrometry

Chromatographic profile of volatile compounds produced during the storage was obtained for LS fish stored at -2°C (Table 3). On the second day of storage, only a few compounds were detected (3-methyl+1-butanol, nonanal and decanal) and TMA was absent but their quantities increased during the storage period. TMA was produced in largest amounts at later stages while substances such as 3-hydroxy-2-butanone (acetoin) were in relatively high quantities in both air and MAP samples. Ethyl acetate was also produced in high quantities but only in the air storage. Other volatile compounds were detected in lower amounts and are summarised in Table 3 according to their retention indices.

**Table 3:** Volatile compounds detected in LS cod loins stored at -2°C as influenced by storage time.

Compound	**RI DB-5ms**^**1**^	Air^2^			MAP^2^		
		Days of storage			Days of storage		
		2	13	23	13	22	28
TMA	< 200	-	8.6	210.7	8.4	96.6	76.2
acetic acid	213	-	0.7	3.5	-	4.3	4.5
2-butanone	214	-	-	-	1.3	-	-
ethyl acetate	221	-	-	79.3	-	-	0.5
2-methyl-1-propanol	229	-	-	12.4	-	5.2	6.4
3-methyl-butanal	246	-	0.3	0.9	-	-	1.3
1-penten-3-ol	263	-	-	1.2	-	2.3	1.8
3-pentanone	271	-	-	2.4	6.8	-	-
S-methyl ester ethanethioic acid	279	-	-	1.7	-	-	-
3-hydroxy-2-butanone	288	-	7.3	47.7	12.8	48.2	58.8
3-methyl-1-butanol	309	0.1	2.3	30.6	1.2	7.4	11.9
2, 3-butanediol	366	-	-	0.5	-	-	
Hexanal	394	-	-	-	-	0.8	0.8
Nonanal	705	1.4	0.3	-	2.3	-	1.8
Decanal	809	0.6	-	1.4	-	1.4	0.9

^1^Calculated ethyl ester retention index (RI) on DB-5ms capillary column

^2^Expressed as PAR (peak area ratio) as determined by GC-MS.

## Discussion

Molecular analyses on bacterial communities in food have only been applied for few years [22,23]. This paper describes the bacterial population developments during storage of cod loins at chilled and superchilled temperatures using both cultivation and cultivation independent approaches.

The molecular methods showed that the flora was directed towards the dominance of *P. phosphoreum*. More diversity was generally seen in early storage in air while during late storage, all samples showed a similar bacterial composition dominated by *P. phosphoreum*. The PCA analysis of t-RFLP patterns indicated that the higher salt content of air-stored products contributed to a different dominating bacterial flora as compared to other treatments since those samples were plotted outside the core pattern in the PCA analysis (Fig. 4). *P. phosphoreum *was found to be below the detection level at the beginning of the storage trial, but as soon as the fish had been stored for 6-7 days, it showed an abundance of 53-100% among the population by 16S rRNA cloning analysis. Cultivation generally showed higher proportional levels of *Pseudomonas *spp. than *P. phosphoreum *in all storage conditions with few exceptions. It has been shown with cultivation that MA packaging enabled *P. phosphoreum *growth in fish products [12] while other bacterial species can dominate as well during air storage [1,16]. The present study confirms its abundance in MA conditions and its ability to dominate under aerobic environmental condition. *P. phosphoreum *has been shown to be able to reach high numbers in aerobically stored fish such as cod, squid and haddock [1,16,17]. In previous shelf life studies on cod and haddock from Iceland, *P. phosphoreum *counts have most often been higher than *Pseudomonas *spp. counts [1,16] but that was not the case in this study.

Discrepancy between PCR strategies and cultivation is a known phenomenon where both approaches are subjective to some degree of bias [24]. Cultivation can be biased to some extent because of different optimal growth conditions and competition between bacterial species in the culture medium, and importantly due to the presence of viable but non-cultivable cells [25]. The Malthus conductance method is based on other principles than colony counts on agar media and the harsh condition (100% CO_2_) of the *P. phosphoreum *method might underestimate their quantity in superchilled, MAP products. As shown in this study, superchilled condition delays the growth rate of *P. phosphoreum *and this effect is enhanced under MA (~50% CO_2_). It is therefore likely that some *P. phosphoreum *cells from these superchilled products may be partly damaged or in such a physiological state that it prolongs the lag phase and/or slows down the growth rate, hence prolonging detection time and giving lower counts during the Malthus incubation. With the molecular approaches, the bias can be derived from the 16S rRNA copy numbers per bacterial genome. Data on 16S rRNA copy number in the *P. phosphoreum *genome is not available but its close relative, *P. profundum *contains 15 copies while *Ps. fluorescens *and *Sh. putrefaciens *contain 5 and 8 copies, respectively (insilico.ehu.es, accessed in april 2008). Other factors such as different DNA extraction efficiency on different species or incongruity in the "universal" priming sites can also influence the outcome [26,27].

The microbiological activity in a fish muscle ultimately leads to its spoilage where different bacterial species contribute to the process in different ways. *Pseudomonas *spp. produce NH_3_, esters and sulphur compounds, *Sh. putrefaciens *produces TMA, H_2_S, hypoxantine and other sulphur compounds and *P. phosphoreum *is able to produce hypoxantine, alcohols, TMA and ketones in particular acetoin [8,9,28]. These organisms are often found in small quantities in newly processed fish but typically reach high numbers during storage. Comparing the composition of microbial species and of volatile compounds it is evident that TMA is the most abundant volatile in mid- and late storage (Table 3) which supports the dominance of *P. phosphoreum*. Acetoin was also detected which can be linked to the presence of *P. phosphoreum *[29]. *Pseudomonas *spp. and *Sh. putrefaciens *have been found responsible for the formation of volatiles sulfides, alcohols (3-methyl-1-butanol, 1-penten-3-ol) and ketones (2-butanone) [30] but these volatiles were in low quantities compared to TMA and acetoin in cod loins which is in agreement with earlier studies on cod fillets [9].

The composition of the natural bacterial flora of a newly caught fish is dependent on its origin and season [31]. Therefore it could be expected that *P. phosphoreum *is more likely to dominate the microflora of fish in Northern seas than from warmer areas. Nevertheless, detection and importance of *P. phosphoreum *in some Mediterranean MA-packed fish products have been reported [12]. The natural flora in the epidermis mucosa of newly caught North-Atlantic cod has been characterised using 16S clone analysis, revealing *Photobacterium*, *Psychrobacter*, *Pseudomonas*, *Acinetobacter*, *Pseudoalteromonas*, and *Flavobacterium *among the commonly found species on cod epidermis [31]. It was reported that *Psychrobacter *spp. was the most abundant species of a 16S rRNA clone library followed by *Photobacterium *spp. in cod caught in the Baltic, Icelandic and North Seas. The bacterial flora of farmed cod from Norway was recently assessed using PCR denaturing gradient gel electrophoresis (DGGE) and it was shown that *Photobacterium *spp., *Sh. putrefaciens *and *Pseudomonas *spp. dominated in MA and air while *Pseudomonas *spp. were solely in dominance in oxygen enriched atmosphere during storage [23]. However, in salt-cured cod the dominating bacteria was found to be *Psychrobacter *spp., representing more than 90% of the bacterial flora [32].

Other bacterial species detected in the study have been isolated and identified from various sources. *Janthinobacterium lividum *is an aerobic bacterium commonly isolated from the microbiota of soils and water of rivers, lakes and springs [33]. The importance of *Flavobacterium *in fish spoilage has not been reported and they are usually overgrown by *Pseudomonas *spp. as shown in fish spoilage model systems [34]. *Flavobacterium *subspecies have been found in other fish species such as catfish and some are also the causative agent of bacterial cold water disease and rainbow trout fry syndrome [35,36]. *Sphingomonas *spp. have been identified in marine waters and in meat processing plants at high levels with molecular based identification [37,38]. *Sphingomonas *and *Variovorax *have also been isolated from deep sea sediments [39]. *Moritella *spp. have been found in marine fish, e.g. *Moritella viscosa *which is a fish pathogen [40].

## Conclusion

The present work describes an investigation of the succession of the dominating bacterial species during storage of unbrined and brined cod loins at chilled and superchilled temperatures under aerobic and MA conditions. Cultivation on selective media indicates a slight dominance of *Pseudomonas *spp. in air-stored samples at low temperatures while molecular based methods, both 16S rRNA cloning analysis and t-RFLP, indicate a high dominance of *P. phosphoreum *in both air and MA packaging. Analysis of volatiles produced during storage at -2°C supported the dominance of *P. phosphoreum *showing intense TMA production. The species diversity was higher after short storage of less than one week, especially in air packaging, but with time, *P. phosphoreum *reached a high dominance, depending on the storage conditions. Discrepancy was observed between the conventional cultivation and molecular methods and requests a further investigation to elucidate this matter. Nevertheless, combined strategy of cultivation and cultivation independent methods might be the key for deeper understanding of bacterial population developments during the spoilage process of food.

## Methods

### Raw material

The fish used for the shelf life experiments was captured by trawl in October 2006 in the North of Iceland, gutted onboard, washed with excessive seawater and stored iced in tubs until filleted, providing a temperature around 0°C. The sea temperature was 8.5-9°C on the day of capture. The raw material was 2-3 or 4-5 days old when it was filleted, deskinned, cut into loins and packaged for the shelf life experiment.

### Storage conditions

Earlier to packaging, a part of the fish was filleted and stored in 4% brine for two days at around 1°C while the other part was processed and cooled down in a 4% brine for 8 min prior to trimming and packaging. These treatments resulted in two groups with a final salt (NaCl) concentration of 2.5 ± 1.0% (HS) and 0.4 ± 0.2% (LS). The fish was stored in air (open bags in styrofoam boxes) and in modified atmosphere packaging (50% CO_2_, 5% O_2_, 45% N_2_) at 0°C (only LS group), -2°C and -4°C resulting in 10 treatments (Table 4). Temperatures were monitored with loggers placed in packages at the bottom recording the temperature every 90 s. The gas composition was monitored using a CheckMate 9900 instrument (PBI Dansensor, Ringsted, Denmark). Sampling was performed in duplicate periodically during the storage time. Aerobic samples were stored for 12 (0°C) and 15 days (-2°C), MA-packed samples at 0°C for 21 days but 28 days for superchilled products.

**Table 4:** Overview of fish treatments tested

Treatments	Temperature (°C)	Atmosphere	Salt content	Sampling time (days)
1	0	Air	LS	6, 13
2	-2	Air	LS	6, 15
3	-4	Air	LS	6, 15
4	0	MAP	LS	7, 21
5	-2	MAP	LS	7, 28
6	-4	MAP	LS	7, 21
7	-2	Air	HS	6, 15
8	-4	Air	HS	6, 15
9	-2	MAP	HS	13, 21
10	-4	MAP	HS	7, 28
R	Raw material			0

### Cultivation

Viable microbial developments were done essentially as described before [16]. Presumptive *Pseudomonas *were cultivated on modified Cephaloridine Fucidin Cetrimide (CFC) agar [41], H_2_S-producing bacteria on iron agar (IA), *Photobacterium phosphoreum *with Malthus conductance method [42] and total viable psychrotrophic counts on Long and Hammer's medium (LH) [43]. An estimate of relative abundance of specific bacterial groups in samples was calculated by dividing their count on specific medium by that of total viable count (LH) of each respective sample. This was done to compare the relative abundance of cultivated bacteria to those obtained via 16S rRNA analysis.

### DNA extraction

During the shelf life trials, fractions of tenfold diluted fish samples were collected and kept at -80°C until DNA extraction. Raw material and 20 storage trial samples were selected for 16S rRNA analysis. Template genomic DNA was isolated from one ml of these diluted samples as described before [44]. The sample was centrifuged at 11000 × g for 7 min to form a pellet. The supernatant was discarded and DNA was recovered from the pellet using Promega Magnesil KF, Genomic system (MD1460) DNA isolation kit (Promega Corporation, Madison, USA) in combination with KingFisher magnetic beads automatic DNA isolation instrument (Thermo Labsystems, Waltham, USA).

### 16S rRNA analysis

The raw material and two samples from each treatment were selected for DNA analysis, from early storage (days 6-7) and late storage (13-15 in air samples and 21-28 in MA samples) resulting in a total of 21 samples. The PCR reaction was done by amplifying the 16S rRNA gene with universal primers, 9F and 1544R (5'-GAGTTTGATCCTGGCTCAG-3 and '5-CCCGGGATCCAAGCTTAGAAAGGA-3' respectively). PCR reaction conditions, cloning and sequencing of the PCR products obtained from the cod samples was performed essentially as described before [45]. Sequencing was performed directly after the PCR reaction. Partial sequencing was performed with R805 primer; '5-GACTACCCGGGTATCTAATCC-3' resulting in 500-600 bp read length. The species coverage by the 16S analysis was estimated using the equation

where C is coverage, n1 is the number of unpaired sequences (number of sequences that did not group with any other in the annealing) and Nt is the number of total clones analyzed. Multiple alignments were carried out using ClustalW (v.1.83) and subsequent phylogenetic dendrogram of the 16S rRNA was plotted with the neighbour-joining software using NjPlot.

### Terminal restriction fragment length polymorphism (t-RFLP)

Extracted DNA from duplicate samples was pooled prior to PCR for the t-RFLP analysis. The PCR was performed with 9F forward primer (sequence above) with a 5' FAM terminal label and HEX labelled reverse primer 805R. The labelled PCR products were digested with *Hae*III and *Alu*I (Fermentas, Hanover, MD, USA) in a 10 μL reaction volume for 2 h. The digested PCR product was diluted 1:20 and 2 μL added to 8 μL of GeneScan 500 LIZ internal size standard (Applied Biosystems, Warrington, UK) in formamide. The fragment analysis was carried out in ABI3730 DNA analyzer. A peak in the chromatogram, here after called terminal restriction fragment (t-RF), is regarded as one taxonomic unit. Data analysis was carried out on the GeneMapper software (v.4.0) using the AFLP analysis method. Peaks below a fluorescence threshold level of 50 were excluded except where a clear trend of same t-RF was detected in other samples. Estimation of relative quantity of *P. phosphoreum *was done by calculating the ratio of its peak area to the total peak area generated in the chromatogram.

### Statistical analysis of t-RFLP profiles

The relative abundance of each t-RF in the profile was calculated by dividing the respective peak area of each t-RF with the total peak area generated between 50-600 base pairs. The profiles from different combinations of labelled primers and restriction enzymes were all combined in one dataset for principal component analysis (PCA) to enhance the analytical power of the model. PCA of t-RFLP profiles from different fish samples was performed using the Unscrambler version 9.5 (Camo ASA, Oslo, Norway). The data was not weighed in order to maintain the ability of t-RFLP to quantitatively discriminate between peaks, representing different taxonomic units. Full cross validation was used.

### Gas Chromatography-Mass Spectrometry

Air and MAP LS samples stored at -2°C were analysed at the beginning, mid- and at the end of storage. About 175 g of fish fillets were cut in pieces and dispersed evenly on a sampling dish (plastic tray). Measurements and identification of volatiles was done according to Olafsdottir et al. [9].

## Authors' contributions

ER carried out the molecular genetic studies, participated in storage trial and drafted the manuscript. HLL was in charge of experimental design of the storage trial, performed all *P. phosphoreum *measurements using the Malthus method and contributed significantly to the manuscript preparation. HM was in charge of the cultivation procedures during the storage trials. RJ and GÓ conducted the GC-MC assays and participated in the design of the study. VTM and GÓH were involved in the design of the molecular genetics work and contributed significantly to the manuscript preparation. All authors read and approved the final manuscript.
